# Plant grafting: new mechanisms, evolutionary implications

**DOI:** 10.3389/fpls.2014.00727

**Published:** 2014-12-17

**Authors:** Eliezer E. Goldschmidt

**Affiliations:** The Robert H. Smith Institute of Plant Sciences and Genetics in Agriculture, Faculty of Agriculture, Food and Environment, The Hebrew University of JerusalemRehovot, Israel

**Keywords:** epigenetics, genome transfer, graft hybrids, incompatibility, long-distance signals, microRNA

## Abstract

Grafting, an old plant propagation practice, is still widely used with fruit trees and in recent decades also with vegetables. Taxonomic proximity is a general prerequisite for successful graft-take and long-term survival of the grafted, composite plant. However, the mechanisms underlying interspecific graft incompatibility are as yet insufficiently understood. Hormonal signals, auxin in particular, are believed to play an important role in the wound healing and vascular regeneration within the graft union zone. Incomplete and convoluted vascular connections impede the vital upward and downward whole plant transfer routes. Long-distance protein, mRNA and small RNA graft-transmissible signals currently emerge as novel mechanisms which regulate nutritional and developmental root/top relations and may play a pivotal role in grafting physiology. Grafting also has significant pathogenic projections. On one hand, stock to scion mechanical contact enables the spread of diseases, even without a complete graft union. But, on the other hand, grafting onto resistant rootstocks serves as a principal tool in the management of fruit tree plagues and vegetable soil-borne diseases. The ‘graft hybrid’ historic controversy has not yet been resolved. Recent evidence suggests that epigenetic modification of DNA-methylation patterns may account for certain graft-transformation phenomena. Root grafting is a wide spread natural phenomenon; both intraspecific and interspecific root grafts have been recorded. Root grafts have an evolutionary role in the survival of storm-hit forest stands as well as in the spread of devastating diseases. A more fundamental evolutionary role is hinted by recent findings that demonstrate plastid and nuclear genome transfer between distinct *Nicotiana* species in the graft union zone, within a tissue culture system. This has led to the formation of alloploid cells that, under laboratory conditions, gave rise to a novel, alloploid *Nicotiana* species, indicating that natural grafts may play a role in plant speciation, under certain circumstances.

## INTRODUCTION

Grafting is an ancient, vegetative, asexual plant propagation technique. It is accomplished most commonly by connecting two plant segments, the shoot piece known as ‘scion’ and the root piece called ‘rootstock’(stock). A broad range of classical grafting techniques can be found in Garner (2013); recent seedling micrografting protocols have been summarized by Turnbull (2010). Grafting has been practiced for many centuries with perennials – mainly fruit trees but also some forest trees and ornamentals – but, as of the early 20th century also with vegetable crops, mainly *Cucurbitae* and *Solanaceae* species. Thus, the majority of recent grafting research concerns physiological and pathological aspects of vegetable grafting. Grafting also plays an important role in various types of physiological investigations (Lifshitz et al., 2006; Omid et al., 2007; Wang, 2011), in particular in classical studies on the movement of the floral stimulus (Zeevaart, 2006). An overview of the current state of the art can be obtained from several recent reviews, each emphasizing a slightly different aspect (Pina and Errea, 2005; Aloni et al., 2010; Harada, 2010; Louws et al., 2010; Martinez-Ballesta et al., 2010; Koepke and Dhingra, 2013). The history of grafting has been described in detail by Mudge et al. (2009). Attempts to provide adequate explanations for the immediate and long-term effects of grafting have been made for generations but, according to a recent review, this plant propagation practice is still shrouded in mystery (Koepke and Dhingra, 2013). Undoubtedly, the use of a large number of diverse plant species in grafting studies has slowed down progress in this research area. The recent introduction of the *Arabidopsis* model into grafting research (Turnbull et al., 2002; Turnbull, 2010) has opened the way for more targeted, advanced grafting research. Thus, the purpose of the present, concise update is threefold: (a) To point out a few major lacunas in our understanding of grafting processes. (b) To introduce some novel, emerging graft physiology mechanisms. (c) To discuss recent evidence that suggests a role for natural grafting in plant evolution.

**FIGURE 1:**
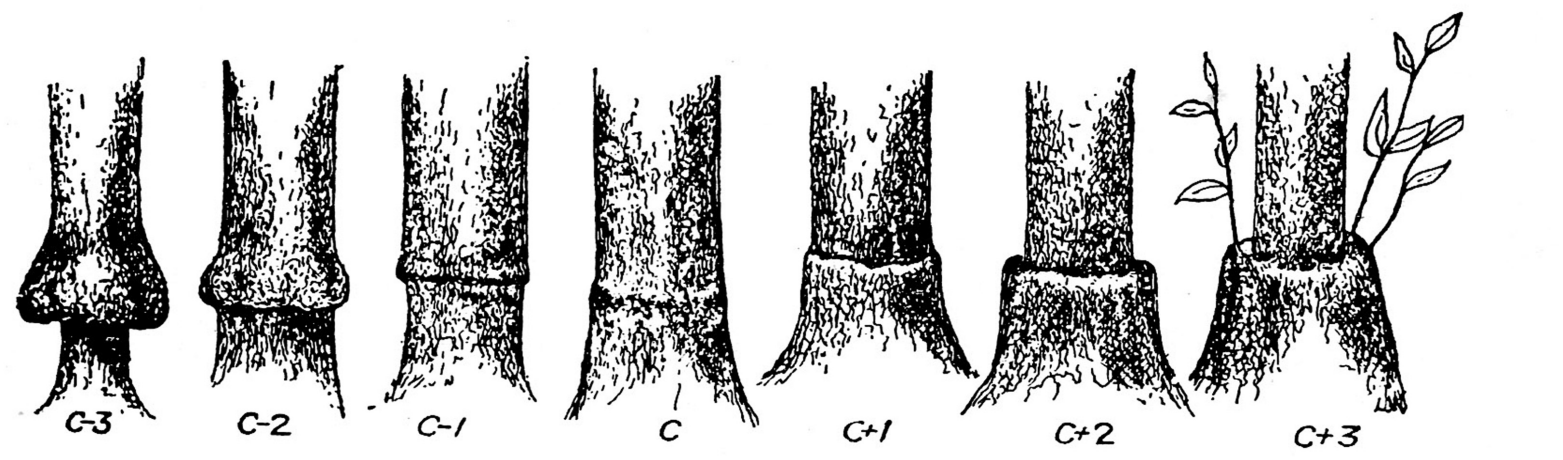
**A range of citrus graft union shapes, presumably indicating rootstock-scion compatibility (reproduced from Webber, 1948; with permission)**.

## GRAFT COMPATIBILITY AND INCOMPATIBILITY

There is no precise definition of ‘graft compatibility’; it generally means establishment of a successful graft union as well as extended survival and proper functioning of the composite, grafted plant. Taxonomic affinity is a prerequisite for graft compatibility. Homografts (= autografts) are presumably always compatible. In heterografts, broadly speaking, intraspecific grafts (= rootstock and scion belonging to the same botanical species) are nearly always compatible, interspecific grafts (= rootstock and scion belonging to different species of the same genus) are usually compatible, intrafamilial grafts are rarely compatible, and interfamilial grafts are essentially always incompatible (Mudge et al., 2009). Examination of heterografts compatibility should include homograft controls (Olmstead et al., 2006; Flaishman et al., 2008; Kawaguchi et al., 2008), a requirement not always fulfilled (Guan et al., 2012). Even in homografts, where rootstock and scion share the same genetic background, certain grafting effects are evident. Differences in rootstock and scion age and juvenility markedly affect their microRNA (miRNA) expression patterns and reproductive development (Poethig, 2009; Wang et al., 2011; Tzarfati et al., 2013). In sweet pepper (*Capsicum annuum* L.), old-stage homografts (58 days old plants) had slower graft-take than young-stage (34 days) homografts. Old-stage grafts had a lower percentage of xylem connections and seemed to suffer from drought stress (Johkan et al., 2009). The time needed for the establishment of a functional graft union is considerably shorter in herbaceous plants than in trees. In the recently introduced *Arabidopsis* micrograft system 7–12 days are required (Turnbull, 2010) but Yin et al. (2012) achieved functional vasculature in *Arabidopsis* within 3 days after grafting. Several months are commonly required for completion of a functional union in tree grafts (e.g., Olmstead et al., 2006).

Graft incompatibility may be defined as failure to form a successful graft union. Yet, despite innumerous follow-ups of graftage in various types of plants, the reasons for graft incompatibility are still vague. Initial healing of the graft union does not in itself ensure long-term compatibility. In cucurbits, apparently successful grafts proved incompatible 25 days after grafting (Edelstein et al., 2004; Aloni et al., 2008). In certain tree stock/scion combinations incompatibility may appear only after several years. (Mosse, 1962; Wutscher, 1979; Tuttle and Gotlieb, 1985). Although incompatibility is not a measurable quantitative trait, various degrees of incompatibility may be discerned, from mild interference with the normal development of the composite plant to mortality of the stock, scion or both.

As already pointed out by Moore (1984), as yet there is no evidence for a specific biochemical-immunological recognition/rejection mechanism between the graft components. This is in contrast to pollination incompatibility, where specific mechanisms have been identified (Kao and Huang, 1994; Takayama and Isogai, 2005; De Franceschi et al., 2012). Yet, heterograft incompatibility clearly increases with genetic distance (Schöning and Kollmann, 1997; Flaishman et al., 2008), indicating some kind of physiological rejection. On the other hand, incompatibility occurs even among related genera of the same family in a rather unpredictable fashion. Thus, within the *Solanaceae*, reciprocal grafts of tomato (*Solanum lycopersicum* L.) and pepper were considered severely incompatible, whereas tomato and eggplant (*Solanum melongena* L.) only moderately so, in comparison with compatible tomato homografts (Kawaguchi et al., 2008). Considerable variation in degree of compatibility was evident among grafts of melon (*Cucumis melo* L.) onto 22 *Cucurbitae* rootstocks (Edelstein et al., 2004) and among other *Cucurbitae* species (Lee and Oda, 2003) and among chestnut (*Castanea*) species (Huang et al., 1994). In citrus trees, visual inspection of trunks at the graft union was believed to reflect the degree of compatibility (**Figure 1**, reproduced from Webber, 1948), although some graft combinations proved to be successful despite the unsmooth graft union (Wutscher, 1979). Anatomical follow-ups of heterograft unions invariably disclose mild to severe interferences with the formation of a fully functional stock/scion continuum. Changes in vascular anatomy were evident in grafts of apple (*Malus domestica* Borkh. and sweet cherry (*Prunus avium* L.) onto dwarfing rootstocks (Soumelidou et al., 1994; Olmstead et al., 2006) and even in compatible graft unions among *Pinus* species (Darikova et al., 2013). A commonly observed disturbance is a convolution of the vascular elements orientation (Soumelidou et al., 1994; Flaishman et al., 2008; Kawaguchi et al., 2008). Soumelidou et al. (1994) proposed that wound-induced changes in the normal flow of endogenous auxin, which plays a key role in vascular differentiation (Aloni, 1995; Cano-Delgado et al., 2010), might be responsible for this distortion. This hypothesis is supported by the recent study of Yin et al. (2012) who demonstrated the involvement of auxin in early stages of graft union formation. The oxidative stress symptoms reported by Aloni et al. (2008) appear only at a considerably later stage and may represent a belated response to the auxin imbalance.

## ROOT–TOP INTERACTIONS

Major grafting effects are readily comprehensible if viewed in the broader context of root–top relations. Thus, dwarfing rootstock effects (Webster, 2002) are not surprising once the well-documented Bonsai culture and root restriction effects are kept in mind (Erez et al., 1992; Ismail and Davies, 1998). By the same token, the invigorating effect of strong, expansive *Cucurbitae* rootstocks on their scions (Blestos, 2005; Martinez-Ballesta et al., 2010) might be anticipated, although the precise physiological mechanisms involved need clarification. The rather complex natural root–top relationship is further complicated in the composite, grafted plant, which undergoes a drastic wounding/healing operation, followed by life-long interactions between different genomes.

Most long-term compatibility studies have been conducted with fruit trees. Xylem graft union anatomy determines the hydraulic root–top conductivity in apple, thereby affecting the growth potential of rootstock/scion combinations (Atkinson et al., 2003). Phloem graft union irregularities appear to be a major source of long-term incompatibility. The pioneering studies of Gur et al. (1968) demonstrated a gradual build-up of biochemical poisoning reaction between pear (*Pyrus communis* L.) scions and quince (*Cydonia oblonga* Mill.) rootstocks. Prunasin, a cyanogenic glucoside, rises from the quince rootstock into the pear scion, where it is catabolized enzymatically by pear glycosidase to liberate cyanide at the graft interface. Cyanide ‘poisons’ the graft union tissues, causing cellular necrosis at the graft interface, leading to severe incompatibility (Gur et al., 1968). However, further studies with peach (*Prunus persica* L. Batsch) grafted on myrobalan plum (*Prunus cerasifera* L. Ehrh) suggested a more general poisoning mechanism, due to progressive impairment of the phloem carbohydrate transport and accumulation of starch above the graft union (Breen and Muraoka, 1975; Moing et al., 1990). Gradual starch accumulation is indeed a symptom of serious interference with nutrient metabolism and root starvation (Moing and Gaudillere, 1992). These phenomena resemble the damage often caused by girdling (= removal of a ring of bark from the trunk or branch of a tree; Li et al., 2003; Goren et al., 2004). Rootstock-scion interactions persist throughout the life of the composite plant, even where satisfactory graft compatibility has been achieved. The upward supply of water and mineral nutrients as well as the downward flow of photosynthates are modified and so is the root–top interchange of hormonal signals (Cutting and Lyne, 1993; Aloni et al., 2010). These mechanisms may account for many of the well-known grafting effects.

## PROTEIN AND RNA SIGNALS

Long-distance phloem transport of proteins and RNAs and their potential role in inter-organ signaling has become a major research domain in recent years (Golecki et al., 1998; Lough and Lucas, 2006; Omid et al., 2007; Kalantidis et al., 2008; Harada, 2010; Kehr and Buhtz, 2013; Spiegelman et al., 2013). Trans-graft transmission of gene silencing in tobacco (*Nicotiana tabacum* L.), which presumably involves some kind of mobile RNA, was one of the pioneering findings in this area (Palauqui et al., 1997). A number of small RNAs (Buhtz et al., 2008) and even some mRNA (Haywood et al., 2005; Omid et al., 2007; Spiegelman et al., 2013) have been identified in phloem saps. Root-to-top as well as top-to root graft transmissible signals have been convincingly demonstrated (Brosnan et al., 2007; Molnar et al., 2010). The involvement of miRNAs in grafting effects has been demonstrated recently by Tzarfati et al. (2013). Reduction of juvenility is one of the most conspicuous effects of grafting in trees (Wareing, 1959; Mudge et al., 2009). The juvenile-to-adult transition in trees (Wang et al., 2011) as well as in annuals (Poethig, 2009) is mediated by a marked decline in the expression of miR156. Both heterograft and homograft citrus scions had reduced miR156 (and the related miR157) expression rates as compared with non-grafted control seedlings, suggesting a role for miRNAs in primary grafting effects (Tzarfati et al., 2013). Changes in the expression of specific miRNAs have recently been implied in heritable chimeras of grafted *Brassica sp.* (Li et al., 2013); however, only a few miRNAs have been shown to be graft-transmissible so far (Buhtz et al., 2010; Bhogale et al., 2014).

There is no obvious reason why a viable, physiologically functional graft union should interfere with regular, long-distance vascular transport. However, the occurrence of a compound which supposedly originated in one organ (e.g., apical meristem), in a distant organ (e.g., roots), does not in itself prove its vascular transport, since this compound could also be synthesized in the presumably recipient organ. Therefore, grafting of genetically distinct mutants/transgenes is still being used as a principal means to distinguish between the site of biogenesis and the recipient organs (Buhtz et al., 2010; Molnar et al., 2010; Turnbull, 2010). Roots of grafted *Arabidopsis* contained specific small RNAs that originated in the apical, source organs, although they were less abundant than in the source tissue by three orders of magnitude (Molnar et al., 2010). Furthermore, the identification of a mobile macromolecule does not indicate in itself a physiological role. The complexities of this area have been discussed by Melnyk et al. (2011).

Notwithstanding, physiologically meaningful protein and RNA long-distance signaling has been convincingly demonstrated in several plant systems. The identification of the FT protein as a major, graft-transmissible component of the long-sought universal florigen is a highly significant achievement (Corbesier et al., 2007; Zeevaart, 2008). The role of phloem mobile miRNAs in the systemic regulation of several key mineral nutrients appears to be a principal mechanism (Buhtz et al., 2010; Kehr, 2013). The regulation of potato (*Solanum tuberosum* L.) tuberization by the graft transmissible miR156 is another recently studied case (Bhogale et al., 2014). The regulatory role of graft transmissible mRNA has now been clearly demonstrated with the *Gibberellic Acid Insensitive (GAI)* mRNA, including the anticipated changes in plant phenotype (Xu et al., 2013b). Thus, we have by now ample evidence for the regulatory role of both protein and several types of RNA as long-distance, graft-transmissible signals, and further discoveries in this area can be expected.

## PATHOLOGICAL ASPECTS

The role of grafting in plant disease is a “double-edged sword.” On one hand, grafting played a pivotal role in the spread of many important plant diseases. But, on the other hand, grafting has become the principal means in overcoming most hazardous plant epidemics and pests.

Plant pathogens spread in tree communities (forests and plantations) through a variety of tracks, including natural, underground root grafts. This avenue of disease transmission has been underestimated (Epstein, 1978), just as root grafting in general has been somewhat neglected (Lev-Yadun, 2011). Its significance became evident with the burst of oak wilt and Dutch elm fungal disease epidemics during the midst of the 20th century (Epstein, 1978). Also viral diseases are transmitted by root grafting (Fulton, 1966; Epstein, 1978). The frequency of intraspecific and interspecific root grafts in wild forest stands has been examined in just a few cases (Blaedow and Juzwik, 2010). Generally, there are considerable differences in the actual significance of disease spread by root grafts between crops and habitats.

Parasitic plants such as mistletoes (= parasitic plants in the order *Santalales*) and dodder (*Cuscuta sp*.) transmit pathogens very efficiently, even among intergeneric and interfamilial plant species (Birschwilks et al., 2006; Mudge et al., 2009; Mikona and Jelkmann, 2010; LeBlanc et al., 2012). Dodder is often used in experimental studies as a bridging vehicle between distant, graft incompatible plant species. Thus, the parasitic, invasive disease transmission mechanism goes beyond the phylogenetic graft compatibility barriers and these seemingly similar pathogen spread pathways cannot be simply equated (LeBlanc et al., 2012).

Grafting of virus infected plant material is another, dangerous way of disease transmission. Graft-transmission of pathogens does not require a compatible, physiologically functional graft union. Even mechanical contact, such as using a contaminated grafting device, can spread viral and bacterial diseases (Barbosa et al., 2005; Bausher, 2013). Interfamilial grafts of citrus on avocado (*Persea americana* Mill.) transferred a citrus viroid to avocado; the grafted citrus budwood remained viable for almost a year without forming a true graft union (Hadas et al., 1992). Periwinkle (*Catharanthus sp.*) seems to be unique in its ability to form distant interspecific grafts. The apple proliferation phytoplasma disease was transmitted from infected apple scions to periwinkle rootstocks (Aldaghi et al., 2007). There is no evidence, however, that such periwinkle grafts form a true, functional graft union.

The role of grafting in the management and control of a broad array of plant pests is nowadays a high priority topic; grafting of *Cucurbitae* and *Solanaceae* vegetables to control soil-borne diseases is widely practiced (King et al., 2008; Louws et al., 2010). The beginnings are, however, considerably older. Grafting of citrus onto sweet orange to combat the *Phytophthora* foot rot (Wutscher, 1979), and grafting of *Vitis vinifera* onto American (*Vitis labrusca* and other species) rootstocks to overcome the *Phylloxera* grapevine plague (Mudge et al., 2009) were adopted in the 19th century. The 20th century struggle of the citrus industry with the *Citrus tristeza virus* (*Closterovirus*) and other virus-like diseases exploited every possible graft combination (Bar-Joseph et al., 1989). The selection of suitable fruit tree rootstock/scion combinations is dominated by disease resistance considerations even today; soil and climate adaptation also affect the preferable stock/scion selection.

Nevertheless, most recent research focuses on vegetable grafting. Superimposed on the natural plant disease resistance (Hammond-Kosack and Jones, 1997) comes once more the complexity of the composite, grafted plant. The diverse mechanisms involved in disease resistance of grafted vegetables have been reviewed by Guan et al. (2012). These range from genetic non-host resistance and rootstock vigor effects to rootstock-induced systemic acquired resistance, which involves salicylate (Conrath, 2006; Park et al., 2008), jasmonate (Schilmiller and Howe, 2005), and other plant hormone signals (Bari and Jones, 2009; Aloni et al., 2010).

Long-distance protein and RNA signals (see previous section) also seem to play a role in rootstock-induced disease resistance. Polygalacturonase-inhibiting proteins (PGIPs) are plant cell proteins that specifically inhibit the cell wall degrading endo-polygalacturonases of plant pathogens (Aguero et al., 2005). Leaf extracts and xylem exudates from grapevine rootstocks transformed to express the pear fruit PGIP-encoding gene (*pPGIP*) had PGIP activity. PGIP was detected in xylem exudates of untransformed grapevine scions grafted onto transgenic rootstocks expressing *pPGIP,* indicating that the PGIP protein is graft–transmissible. Such scions revealed improved tolerance toward grapevine diseases caused by *Xylella fastidiosa* and *Botrytis cinerea* (Aguero et al., 2005). Following these results, Haroldsen et al. (2012) suggested that cultivars genetically engineered to express disease controlling, graft–transmissible proteins and RNAs, could be used as rootstocks for disease susceptible scion cultivars. Grafting genetically engineered rootstocks with non-transformed fruit tree scions would presumably result in disease-resistant cultivars bearing fruits which are not genetically modified, a desirable combination (Haroldsen et al., 2012). This idea undoubtedly merits further research.

## GRAFT TRANSFORMATION

**FIGURE 2:**
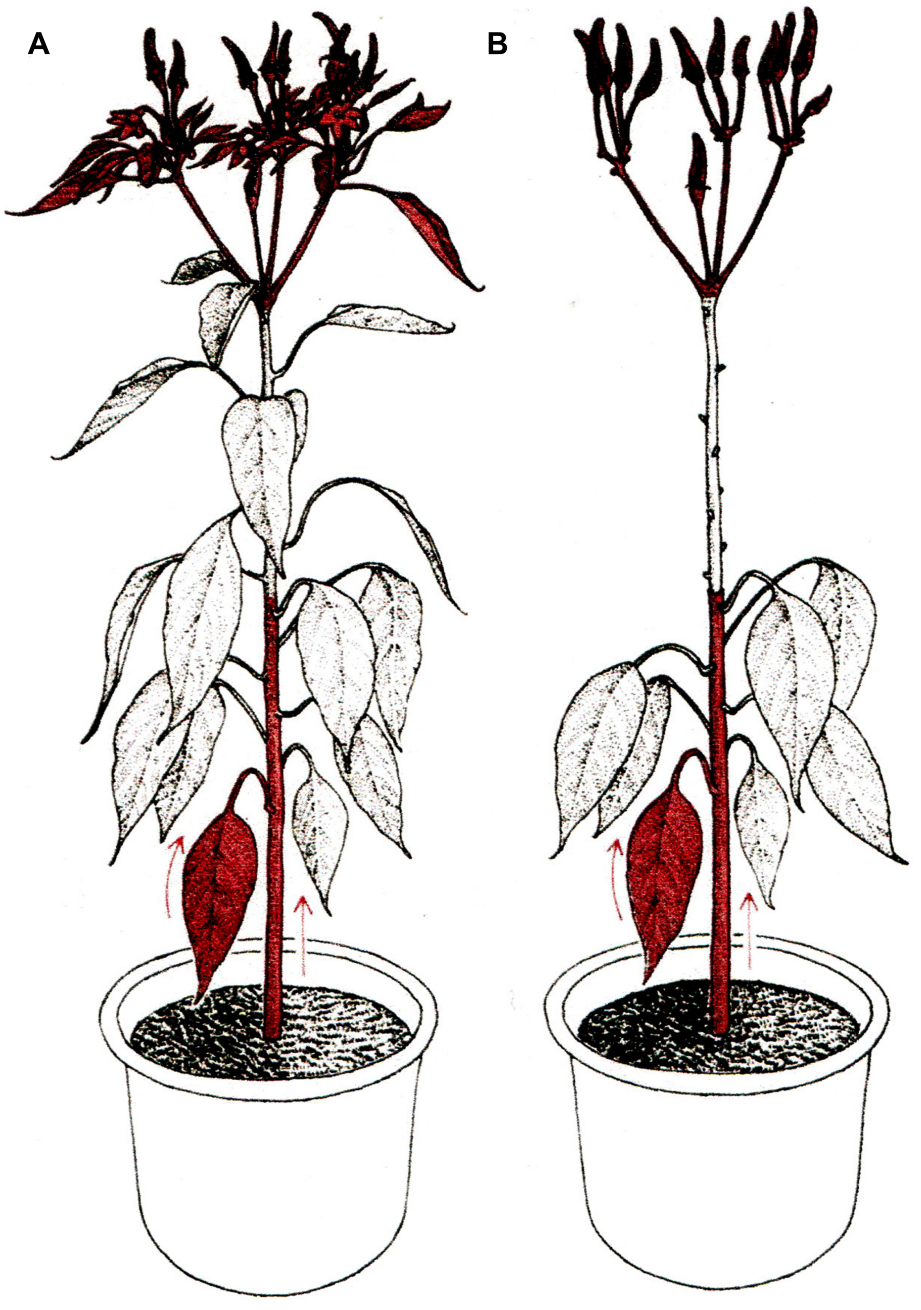
**Mentor grafting. (A)** Non-graft normal plant as control. **(B)** Mentor graft; scion leaves were removed in order to facilitate chromatin translocation from rootstock leaves and stems to the primordial organs of the scion. Arrows indicate the direction of chromatin translocation (reproduced from Ohta, 1991; with permission).

Of all grafting issues, the least understood and most controversial is the ‘graft hybrid’ concept. According to this concept grafting may involve stock to scion transfer of genetic material (= graft transformation), leading to heritable changes in the scion. The scion which has acquired certain heritable traits from the rootstock is regarded as a ‘graft hybrid.’ However, graft transformations occur only under ‘Mentor grafting’ conditions, which presumably enforce the transfer of genetic material from stock to scion (**Figure 2**).

In ‘Mentor grafting,’ young seedlings shoots serving as ‘scions’ are grafted onto mature, flowering plants used as ‘stocks.’ To make sure that the scions fully depend on the stock for the supply of nutrients, leaves of the scions (except for two or three leaves at the top) are removed throughout the experiment. Stock fruits are also removed in attempt to maximize flow of substances from the stock to the scion (Ohta, 1991). Interestingly, the ‘Mentor grafting’ technique is the same as that used in classical flowering research, where removal of leaves from the scion was expected to promote stock to scion movement of the floral stimulus (Lang, 1965).

The graft hybrid concept, which was developed and demonstrated by the Soviet horticulturist Michurin (1949), appeared to be in contrast with Mendelian genetics and was rejected (and almost forgotten) by Western scientists who opposed and distrusted the Soviet biology led by Lysenko. Several published studies (and most probably many unpublished) could not confirm the appearance of graft hybrids (Stubbe, 1954; Bohme, 1957; Topoleski and Janick, 1963; Menda et al., 2006). Yet, reports supporting the occurrence of graft transformations appeared from time to time (Frankel, 1956), and increasingly so in recent years (Li et al., 2013; Tsaballa et al., 2013; Wu et al., 2013; Zhou et al., 2013). Proponents of the graft hybrid concept reviewed the supporting evidence and called for reassessment of the graft transformation hypothesis that has been neglected for several decades (Liu, 2006; Liu et al., 2010).

Within the ‘graft hybrid’ supportive evidence some specific characteristics can be defined. (a) The frequency of the appearance of variant plants is highly variable, sometimes below 1% (Ohta, 1991). Thus, the argument that ‘no variants were found’ must be based on a very large number of replicate grafts. Indeed, some of the ‘negative’ reports used several thousands of grafts (Stubbe, 1954). (b) The graft hybrid experimental evidence rests almost exclusively on intra- and interspecific grafting of *Solanaceae*, in particular pepper (Ohta, 1991; Taller et al., 1998; Tsaballa et al., 2013), which is somehow more amenable than other plant species to rootstock-induced scion transformation. (c) The alleged rootstock to scion transmission of genetic material is the most mysterious part of it all. The initial belief of Michurin (1949) that genes can move between rootstock and scion has been refined by Ohta (1991) who presented histological evidence that masses of chromatin are moving via the vascular system from the older rootstock across the graft union to the apical primordia or flower buds of the younger, mentor-grafted scion. The model of amplified plasmodesmatal macromolecular transport toward apical meristems (Ueki and Citovski, 2005) has been cited in this context (Liu, 2006) but no further evidence in support of this mechanism has been presented.

In their review, Mudge et al. (2009) indicated that the emerging concept of graft-transmissible gene silencing signals may hold the key for a new approach to the graft transformation riddle. Recent research further extends this view in conjunction with probable involvement of epigenetic inheritance mechanisms.

Epigenetics refers to reversible heritable changes in genome function that occur without a change in the DNA sequence and may have morphological, physiological, and ecological consequences (Rapp and Wendel, 2005; Fossey, 2009). Changes in DNA methylation are presumably among the principal, ubiquitous epigenetic mechanisms (Rapp and Wendel, 2005) although their heritability requires further elucidation (Paszkowski and Grossniklaus, 2011). DNA methylation during plant gametogenesis, in particular, appears to involve epigenetic, heritable changes (Takeda and Paszkowski, 2006; Calcaro et al., 2012). Plant DNA methylation has been shown to be regulated by siRNAs; ‘siRNA-mediated epigenetic modification’ is currently an acceptable term (Xu et al., 2013a).

As already discussed, graft-transmissible RNA gene silencing signals have been demonstrated in both the upward (Brosnan et al., 2007) and the downward direction (Molnar et al., 2010). Changes in DNA methylation in the recipient organs have been detected; these changes are regarded as epigenetic modifications (Molnar et al., 2010). Partially heritable, locus-specific alteration of DNA methylation patterns have recently been found in scions of interspecific grafts of *Solanaceae* (Wu et al., 2013). Wu et al. (2013) think that their research paves the way for resolution of the graft hybrid controversy. However, further, rigorous research is desperately needed, in order to unequivocally elucidate the graft hybrid – graft transformation issues.

## EVOLUTIONARY SIGNIFICANCE

Grafting is a natural phenomenon, wide spread between roots of the same tree, neighbor trees of the same species or even, although less frequently, among trees belonging to different species (= interspecific grafting). Root grafting could be induced artificially between potted *Picea abies* trees; a pair of compressed roots fused and formed a complete union within several months (Fischer et al., 1960). Natural grafting is not confined to roots; intra- and interspecific grafts among tree branches have been described (Mudge et al., 2009; Stegemann et al., 2012). Although anatomical evidence is scarce (Rao, 1966), it may be assumed that mutual pressure between two adjacent roots builds-up as they increase in diameter, since the surrounding soil precludes much lateral displacement of the two roots. With increasing pressure the bark of each in contact with the other wears away, bringing their cambia in contact, leading eventually to a functional graft union. Most aerial grafts occur when a branch from one tree is “caught” in the forked branch of another tree, which is analogous to the compression between adjacent roots (K. W. Mudge, personal communication, with permission).

Assessment of the evolutionary significance of grafting involves discussion at two levels: its benefits for plant survival and its potential role in the formation of new species. The plausible ecological benefits of root grafting have been discussed by Lev-Yadun (2011). The existence of underground graft contacts turns the individual tree into member of a cooperative community which may support each other in a variety of physical, nutritional and reproductive ways (Eis, 1972). Bormann (1966) observed that stumps sometimes persist and continue to grow for years or even decades, when root-grafted to adjacent trees but not when occurring singly, suggesting that nutrients arriving via root grafts from standing neighbor trees are responsible for the persistence of the stump. Trees that lost their crowns in a storm etc. may survive and resume above ground growth and reproduction owing to their root graft cooperation (Mudge et al., 2009; Lev-Yadun, 2011). On the other hand, as already mentioned in a previous section, there is a very real risk of pathogen transmission via natural root grafts, including fungal, bacterial (Lopes et al., 2009), viral and mycoplasmal diseases (Epstein, 1978). In as much as agriculture is nowadays considered a component of the evolutionary arena (Ross-Ibarra et al., 2007; Thrall et al., 2010), grafting also plays a critical role in the survival of thousands of man-selected plant genotypes which are propagated only by grafting and have never existed in natural habitats (Janick, 2005; Goldschmidt, 2013).

Addressing the significance of grafting as a potential evolutionary mechanism must take into account the ancient belief that grafting may give rise to new plant species (Mudge et al., 2009). The ‘graft hybrid’ concept discussed in the preceding section is also related to this belief. Some of the novel species records may be attributed to graft chimeras, which are not true hybrids, but are inheritable (Swingle, 1927; Mudge et al., 2009). Adventitious buds may develop from the graft junction, and the lateral, developing chimeral shoots may retain a stable, heritable intermediate phenotype. Lateral shoots from graft junction wounds occur frequently, and a considerable percentage of those are stable chimeras (Kaddoura and Mantell, 1991).

Recent research has brought us much closer to a potentially real evolutionary grafting mechanism. In their early report Stegemann and Bock (2009) demonstrated exchange of plastid genome material between stock and scion cells in interspecific graft junction zones, later shown to consist of entire chloroplast genome transfer between distinct *Nicotiana* species (Stegemann et al., 2012). Interestingly, in their initial report Stegemann and Bock (2009) state that their data.

“… do not lend support to the tenet of Lysenkoism that ‘graft hybridization’ would be analogous to sexual hybridization. Instead, our finding that gene transfer is restricted to the contact zone between scion and stock indicates that the changes can become heritable only via lateral shoot formation from the graft site.”

However, in a more recent report (Fuentes et al., 2014) Bock and his team come up with a bolder title; “Horizontal genome transfer as an asexual path to the formation of new species.” Tissue cultured plants were grafted, and the tissue very close to the graft junction (some millimeters from the junction) was maintained and screened further in tissue cultures. The results show that entire nuclear genomes were transferred between plant cells in the graft junction zone, leading to the formation of novel, alloploid plant cells, and such events occurred at a considerable frequency. Plants recovered from these cells are real graft hybrids. A fertile, stable hybrid between herbaceous and woody *Nicotiana* species was thus obtained, tentatively named *Nicotiana tabauca*. Still, natural evolutionary formation of new species via this pathway would depend upon natural graft formation and emergence of adventitious lateral shoots, as previously discussed.

The hypothetical possibility of cell fusion graft hybrids has already been mentioned by Swingle (1927; Figure 12). Genome combinations have indeed been achieved in artificial protoplast fusion systems, and interspecific hybrid plants were obtained (Evans et al., 1980; Shepard et al., 1983). However, the recent findings of Bock’s team raise the exciting option that natural grafting may have played an active evolutionary role in plant speciation. The results obtained with *Nicotiana* under tissue culture conditions require further confirmation with other plants in natural settings (Hare, 2014). It is difficult at the present time to estimate the actual contribution of this asexual path to plant evolution. Although seemingly dependent upon a rare sequence of events, this evolutionary niche could have been exploited as a plant survival bypass under extreme selective pressure conditions.

## CONCLUDING REMARKS

While the technology of grafting has advanced tremendously, the long-term survival of grafted, composite plants is still somewhat unpredictable. The complexity of stock/scion interactions attains a new dimension when pathogenic agents enter the scenery. More enigmatic are the graft hybrid conundrum and the broader evolutionary significance of grafting. The extensive horticultural grafting research has not usually addressed these basic questions. The recently adopted *Arabidopsis* model has opened the way for meticulous, in-depth grafting research. The currently available molecular tools are expected to advance our understanding and eventually resolve the long standing grafting mysteries.

## Conflict of Interest Statement

The author declares that the research was conducted in the absence of any commercial or financial relationships that could be construed as a potential conflict of interest.
